# Eosinophils, Eosinophilic Gastrointestinal Diseases, and Inflammatory Bowel Disease: A Critical Review

**DOI:** 10.3390/jcm13144119

**Published:** 2024-07-14

**Authors:** Giulia Migliorisi, Elisabetta Mastrorocco, Arianna Dal Buono, Roberto Gabbiadini, Gaia Pellegatta, Paola Spaggiari, Francesca Racca, Enrico Heffler, Edoardo Vincenzo Savarino, Cristina Bezzio, Alessandro Repici, Alessandro Armuzzi

**Affiliations:** 1 IBD Center, IRCCS Humanitas Research Hospital, Via Manzoni 56, 20089 Rozzano, Italy; giulia.migliorisi@humanitas.it (G.M.); elisabetta.mastrorocco@humanitas.it (E.M.); arianna.dalbuono@humanitas.it (A.D.B.); roberto.gabbiadini@humanitas.it (R.G.); cristina.bezzio@hunimed.eu (C.B.); 2 Department of Biomedical Sciences, Humanitas University, Via Rita Levi Montalcini 4, 20072 Pieve Emanuele, Italy; gaia.pellegatta@humanitas.it (G.P.); francesca.racca@humanitas.it (F.R.); enrico.heffler@hunimed.eu (E.H.); alessandro.repici@hunimed.eu (A.R.); 3 Endoscopic Unit, Department of Gastroenterology, IRCCS Humanitas Research Hospital, 20089 Rozzano, Italy; 4 Department of Pathology, Humanitas Research Hospital, 20089 Rozzano, Italy; paola.spaggiari@humanitas.it; 5 Personalized Medicine, Asthma and Allergy, IRCCS—Humanitas Research Hospital, 20089 Rozzano, Italy; 6 Department of Surgery, Oncology and Gastroenterology, Department of Medical and Surgical Specialties, University of Padua, 35122 Padova, Italy; edoardosavarino@gmail.com

**Keywords:** inflammatory bowel disease, eosinophilic gastrointestinal disease, eosinophilic esophagitis, eosinophils

## Abstract

Background/Objectives: Inflammatory bowel disease (IBD) and eosinophilic gastrointestinal diseases (EGIDs) are complex, multifactorial chronic inflammatory disorders affecting the gastrointestinal tract. Their epidemiology, particularly for eosinophilic esophagitis (EoE), is increasing worldwide, with a rise in the co-diagnosis of IBD and EGIDs. Both disorders share common risk factors, such as early exposure to antibiotics or specific dietary habits. Moreover, from a molecular perspective, eosinophilic infiltration is crucial in the diagnosis of eosinophilic disorders, and it also plays a pivotal role in IBD histological diagnosis. Indeed, recent evidence highlights the significant role of eosinophils in the health of the intestinal mucosal barrier and as mediators between innate and acquired immunity, even indicating a potential role in IBD pathogenesis. This narrative review aims to summarize the current evidence regarding the common clinical and molecular aspects of EGIDs and IBD and the current state of knowledge regarding overlap conditions and their pathogenesis. Methods: Pubmed was searched until May 2023 to assess relevant studies describing the epidemiology, pathophysiology, and therapy of EGIDs in IBD. Results: The immune pathways and mechanisms underlying both EGIDs and IBD remain partially known. An improved understanding of the role of eosinophils in overlapping conditions could lead to enhanced diagnostic precision, the development of more effective future therapeutic strategies, and a more accurate prediction of patient response. Consequently, the identification of red flags indicative of an eosinophilic disorder in IBD patients is of paramount importance and must be evaluated on a case-by-case basis.

## 1. Introduction

Inflammatory bowel diseases (IBDs) and eosinophilic gastrointestinal diseases (EGIDs) are multifactorial chronic inflammatory disorders of the gastrointestinal (GI) tract. Their epidemiology is increasing worldwide, particularly in newly industrialized countries, placing a great burden on the healthcare system [1,2]. IBDs, encompassing Crohn’s disease (CD) and ulcerative colitis (UC), are mainly characterized by abdominal pain, fatigue, diarrhea, and rectal bleeding. These disorders are typically managed with immune-modulating drugs, although in severe cases and complications, surgery may be required [3,4,5]. EGIDs are further distinguished into eosinophilic esophagitis (EoE), the most prevalent disease of this group, and non-EoE-EGIDs. Non-EoE-EGIDs are less common and include eosinophilic gastritis (EoG), eosinophilic enteritis (EoN) and eosinophilic colitis (EoC) [6]. EoE is a type 2 associated inflammatory disorder characterized by eosinophilic infiltration of the esophageal wall, which may lead to fibrosis and dysfunction of esophageal function in some patients [7]. This results in adult patients with dysphagia, food bolus impaction, chest pain and heartburn. Management includes elimination diets, conventional medications such as proton pump inhibitors and swallowed topical corticosteroids (STCs) or biologic drugs targeting interleukin 4 (IL-4) (e.g., Dupilumab), and endoscopic dilation in cases of fibrotic stenosis [8]. EGIDs and IBD share certain similarities in terms of potential etiologies and risk factors, and they may coexist simultaneously in the same patient [9]. The pathogenesis of both conditions remains unclear, although it is widely acknowledged that they result from the interaction between genetic predisposition, environmental factors, and alterations in the gut microbiota, which can lead to an aberrant immune response and chronic inflammation of the GI system [10]. It has been demonstrated that early-life exposure to antibiotics during gestation, particularly during the first years of life, is associated with an increased risk of developing both EGIDs and IBD, with a particular association with Crohn’s disease in adulthood [11,12,13]. Furthermore, a recent meta-analysis has demonstrated that exclusive breastfeeding confers protection against the development of both IBD and EoE [14,15]. Moreover, dietary habits are considered relevant environmental risk factors for both disorders and, most importantly, specific diet restrictions are paramount in treating EGIDs. Indeed, high-fat diets and ultra-processed foods are associated with an increased risk of developing CD [16,17]. Furthermore, as previously stated, food elimination diets that avoid specific food allergens (e.g., milk, gluten, soy, fish, tree nuts/peanuts, eggs) represent a primary therapeutic approach for EoE, particularly in the pediatric population [18]. Additionally, dysbiosis and the reduction of microbiota species diversity have been reported to impact on the origin and maintenance of both inflammatory disorders [19,20,21]. The role of dysbiosis in the pathogenesis of these diseases may explain the higher risk of developing IBD, especially CD associated with previous PPI use [22,23], which is not observed with other anti-secretory drugs [24]. The interaction between PPIs and EGIDs is controversial, as PPIs are considered a first-line pharmaceutical therapy together with STCs; however, the resulting interference in peptic digestion may increase esophageal exposure to food allergens [25]. Furthermore, from a molecular point of view, the upregulation of shared inflammatory molecules, such as specific subtypes of Toll-like receptors (TLRs), has been observed in both non-treated EoE and in active IBD [26,27]. Similarly, pro-inflammatory cytokines (e.g., interleukin-5, IL-5), which are involved in the activation and recruitment of eosinophils, are overexpressed in the intestinal mucosa of patients with UC and active CD [28,29].

The aim of this narrative review is to provide a critical summary of the common clinical and molecular aspects of EGIDs and IBD and the role of eosinophils in their pathogenesis and to present the current state of knowledge regarding the overlapping of these conditions.

## 2. Eosinophils in the Gut: Their Role in EGIDs and IBD

Eosinophils are a subtype of polymorphonuclear leukocytes derived from bone marrow pluripotential hematopoietic stem cells under the influence of several cytokines, including interleukin 3 (IL-3), 13 (IL-13), IL-5 and granulocyte macrophage colony-stimulating factor (GM-CSF) [30]. Their presence is not limited to the blood and the hematopoietic organs; indeed, tissue eosinophils can be found under physiologic conditions in the mammary glands, uterus, the non-esophageal part of the gastrointestinal tract, and in adipose tissue [31]. Their physiological distribution is uneven in the GI tract [32], with a greater prevalence in the lamina propria of the small bowel, ileum, and colon and almost absent in the esophagus [32,33]. Historically, their known functions were limited to the protection against parasitic infections and food allergy phenomena [34,35]. Nevertheless, the current evidence, although limited to animal models, indicates that eosinophils play a significant role in the immune homeostasis of the entire digestive system [36]. This is particularly evident in the maintenance of the integrity of the intestinal mucosal barrier, in the interaction with gut microbiota, and in the mediation between type I and II immunity. In this context, Jung et al. [37] and Ignacio et al. [38] observed that eosinophil-deficient mice exhibited a significant decrease in the integrity of the mucosal barrier, major villous architecture abnormalities, and intestinal permeability in response to microbial colonization. Eosinophils play a pivotal role in the survival of plasma cells, as evidenced by the release of proliferation-inducing ligand (APRIL) and interleukin-6 (IL-6) in the bone marrow and inducible nitric oxide synthase (iNOS), lymphotoxin, and interleukin-1β (IL-1β) in the GI system. The aforementioned factors are responsible for the T-cell independent IgA switching class and the development of gut-associated lymphoid tissue (GALT) in Peyer patches [37,39]. Furthermore, eosinophils act as intermediaries between the innate and adaptive immune responses, increasing the number of Th2 cells [40,41] and T-regulatory cells such as Th17 [42]. Moreover, in balanced conditions, eosinophils, in conjunction with mast cells, have bidirectional communication with the enteric nervous system (ENS) [41]. This communication influences contractility and secretory bowel function through the release of vasoactive intestinal peptide (VIP) and substance P [43]. It also stimulates nervous growth and participates in the recruitment of immune cells when activated [41,44].

The recruitment of eosinophils in the intestinal mucosa is mediated by several cytokines (e.g., IL-5, IL-13 and interleukin 33 (IL-33) and chemokines (particularly eotaxines) released by white, endothelial, and epithelial cells of the GI tract. Eotaxines (principally eotaxin 1, followed by eotaxines 2 and 3) are chemokines that bind to eosinophils’ surface receptor CCR3, resulting in eosinophil migration and homing in the lamina propria of the bowel mucosa [45,46]. Eosinophilic infiltration is a prominent histological feature in both IBD [47] and in EGID patients [48]. It has been demonstrated that an upregulation of eotaxines, regardless of disease activity, occurs due to overexpression of CCR3 in UC patients [49]. Furthermore, IL-1 and IL-33 have been identified in both animal and human models of colitis [50,51]. The relationship between eosinophils and UC is well supported by the well-defined assumption that UC is characterized by a disorder of the Th2 immune response rather than the enhanced Th1 profile observed in CD patients [52]. However, eosinophils in surgical specimens of resected ileum of CD patients have been linked to an increased risk of early recurrence [29], while peripheral blood eosinophilia has been shown to predict clinically active disease in pediatric CD patients [53] and has been associated with increased disease severity in both CD and UC [54,55].

Eosinophils are directly responsible for intestinal tissue damage through the degranulation of eosinophil-derived neurotoxin (EDN), eosinophil cationic protein (ECP), major basic proteins (MBP-1 and MBP-2), and eosinophil peroxidase (EPO). ECP is a ribonucleasis protein that provides apoptosis signals [56], while MBPs alter cell membrane functions, leading to increased intestinal permeability in the inflamed intestine [57]. MBP, in conjunction with EDN, may also affect the cholinergic pathways of the ENS, resulting in motility dysfunction [56]. Furthermore, elevated levels of MBP and EDN in the feces have been associated with disease activity in both CD and UC and may serve as additional biomarkers, particularly for detecting early responses to biological and steroid therapies [58,59]. Smyth et al. [60] observed that eosinophils were selectively localized near the ENS nerves in the mucosa, extending to the muscle layer in patients with CD. This resulted in enhanced substance P release and choline acetyltransferase nerve function. Moreover, eosinophils can contribute to the perpetuation of inflammation by synthesizing chemokine CXCL8, which has been observed to be responsible for neutrophil infiltration in the intestinal lamina propria, which is directly related to the severity of disease in UC patients [61]. Finally, recent evidence has shown the potential role of eosinophils in the development of fibrosis. Although eosinophil infiltration has been associated with fibrosis in other districts [57] and an abnormal distribution of eosinophils and overexpression of IL-33 has been observed in CD ileum stricture in a pediatric population [62], more recent studies have demonstrated that eosinophil depletion can protect against chronic inflammation but does not influence collagen deposition and fibrosis development [63].

**Figure 1:**
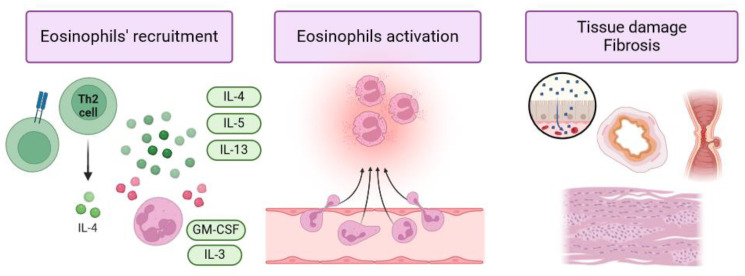
Molecular pathways shared by IBD and EGIDs. Different chemokines and cytokines are involved in eosinophils activation. While IL-3 and GM-CSF are responsible for activation and promoting eosinophils maturation in bone marrow, many other molecules are mainly involved in eosinophils’ recruitment, such as IL-5, IL-4, IL13 and IL-33. The activation and eosinophils’ degranulation can directly cause tissue damage, altering membrane permeability and barrier function. Moreover, the products of eosinophils’ degranulation can alter nervous pathways and cause neural apoptosis with irreversible altered nervous functions of the enteric nervous system. Additionally, eosinophils can induce the deposition of collagen with fibrotic tissue remodeling by the release of tumor-growing factor-β (TGF- β).

EGIDs and specifically EoE are type 2-associated inflammatory disorders that result from the interaction of genetic predisposition, food sensitization and the abnormal infiltration of eosinophil cells in the mucosa layers. Genetic variants and polymorphisms of genes involved in eosinophil homing (e.g., CCL26, eotaxin 3) and eosinophil activation and differentiation in Th2 cells (TSLP, WDR36) have been associated with an increased risk of developing EoE [64]. Chemokines involved in eosinophil migration, particularly eotaxin-3, are overexpressed in the epithelium of EoE patients as well as in IBD patients and may represent a potential therapeutic target [65]. Eosinophil degranulation products directly damage the mucosa layer and mucosal barrier function. Furthermore, in IBD, ECP and MBP can alter membrane permeability and mucosal barrier function, while EDN is involved in the activation and recruitment of dendritic cells, which in turn promotes the production of Th2 profile immune cytokines, such as IL-33, IL-5, IL-13, which sustain the inflammatory process [7,66]. Recent evidence suggests a potential role of eosinophils in esophageal motility disorders observed in EoE. For instance, MBP interferes with the cholinergic nervous pathway, while the release of IL-13 and IL-6 causes relaxation of the esophageal sphincter. Cytotoxic eosinophilic granules can cause neural apoptosis with irreversible alterations to nervous function [67]. In this regard, a recent retrospective study observed esophageal dysmotility and achalasia in almost 15% of EoE patients [68], and a recent systematic review by Visaggi et al. highlighted the resolution of motility disorders in EoE patients on treatment, supporting the hypothesis of an interaction between eosinophils and pathological functions of the ENS [69]. Nevertheless, eosinophils produce transforming growth factor-β (TGF-β), which induces the expression of collagen and fibronectin, resulting in fibrotic tissue remodeling [70]. Another potentially important factor in the pathogenesis of eosinophilic disorders is the microbiota of the gastrointestinal tract. A recent systematic review examined current knowledge regarding the role of the microbiota in EoE pathogenesis. It highlighted similarities between the oral and esophageal microbiota and microbial products, as well as a higher microbiota load in EoE patients compared to controls [71]. Furthermore, Massimino et al. [20] reported a potential correlation between microbe species and alimentary allergens triggering the inflammatory process. Finally, Facchin et al. showed that members of the Actinobacillus, Bergeyella, Porphyromonas and Alloprevotella genera were positively associated with biological samples with eos/HPF > 15 [72]. Figure 1 elucidates the molecular mechanisms possibly shared by IBD and EGIDs.

## 3. Diagnosis and Endoscopy: Differences and Similarities

The accurate assessment of disease severity and prognosis in both EGIDs and IBD is contingent upon a comprehensive description and quantification of endoscopic alterations, complemented by the collection of bioptic samples for diagnostic purposes. In detail, endoscopic scores of activities currently recommended for EoE and EoG (EREFS and EG-REFS, respectively) include description/evaluation of both inflammatory and fibrotic aspects [73]. EoE is endoscopically characterized by the presence of edema, rings, exudates, furrows, and strictures. However, according to the literature, in a variable proportion of patients (5–32%), these typical endoscopic features are not visible in EoE, and the esophageal mucosa may appear normal [74,75]. In contrast, the EG-REFS is based on the presence of erosions/ulcers, raised lesions, fold thickening, and pyloric stenosis or friability, erythema, and granularity of the gastric mucosa [76,77,78,79]. EoC and EoN are typically characterized by a normal mucosa appearance, with non-specific edema, erythematous areas, or aphthous lesions [80].

In UC, endoscopy mostly reveals a homogenous and continuous inflammation that originates in the rectum and extends proximally. The presence of erythema, friability and bleeding of the mucosa, erosions, and ulcers are evaluated and included as core items in the Mayo endoscopic score (MES) and the Ulcerative Colitis Endoscopic Index of Severity (UCEIS). Conversely, CD is characterized by a patchy and transmural inflammatory behavior, often with normal mucosa. Typical endoscopic features of CD include aphthous ulcers and deep serpiginous ulcers, strictures, pseudopolyps, and fistulas. In the evaluation of disease activity and severity, as recommended by the European Crohn’s and Colitis Organization (ECCO), either the Simple Endoscopic Score for Crohn’s Disease (SES-CD) or the Crohn’s Disease Endoscopic Index of Severity (CDEIS) can be adopted [81,82,83,84].

Documenting eosinophilic infiltration is a diagnostic and necessary parameter for EGIDs and is based on an adequate and extensive bioptic sampling (i.e., at least two biopsies from the distal, mid, and upper esophagus in case of suspected EoE) [85]. The diagnosis of EGIDs requires the presence of eosinophilic infiltration above specific thresholds depending on the disease location. The diagnostic threshold for EoE is a peak of at least 15 eosinophils per high-power field (HPF) [85,86,87]. There is no consensus regarding the definition of eosinophil thresholds for non-EoE EGIDs. However, it is widely acknowledged that non-EoE EGIDs have higher eosinophilic infiltration cut-offs. For EoG and EoN, the proposed threshold is greater than or equal to 30 eosinophils per HPF for the stomach, greater than or equal to 50 eosinophils in the duodenum, and greater than 56 eosinophils per HPF in the ileum [88]. Eosinophils are typically absent from the left colon and rectum and are rarely observed in the right colon. In adults, normal values range from 1 to 3 cells per HPF, while in children, they range from 50 to 100 cells per HPF [89]. The pathological threshold for EoC in adults is defined as greater than 40 cells per HPF in at least two colonic segments [90]. Eosinophils in EoC are mainly located in the submucosa and within the crypt epithelium. Such instances are rare and do not result in the formation of crypt abscesses or extensive degranulation, as observed in patients with IBD [91]. If IBD is suspected, it is recommended that at least two biopsies be taken from the terminal ileum and each segment of the colon (cecum, ascending colon, transverse colon, descending colon, sigmoid colon, and rectum) [92]. This is regardless of whether the mucosa appears endoscopically normal [93,94]. In case of suspected involvement of the upper digestive tract, esophageal, gastric, and duodenal biopsies are indicated in patients with known IBD [93]. However, it is important to underline that abnormalities in the upper digestive tract have been described in almost 80% of CD patients of pediatric age [95]. The number of eosinophils present in UC colon samples is variable, but their coexistence with basal plasmacytosis (more than three cells at the base and lateral part of the crypts) increases the likelihood of a correct diagnosis [96]. Furthermore, eosinophilia (more than 60 cells HPF), especially in the left colon at diagnosis, may be predictive of non-response to medical treatment [97,98]. In contrast to UC samples, eosinophils are less characteristic in CD samples. For instance, eosinophils can be observed in conjunction with neutrophils on the surface of the intestinal epithelium in the early CD ileum [99,100]. Conversely, their presence alongside T lymphocytes within the submucosal or intramuscular plexus (ganglionitis) in surgical specimens is a distinctive histological feature of transmural inflammation and a prognostic factor for disease recurrence when present in the resection margins [101]. Esophageal involvement in CD is exceedingly rare and is typically characterized by erosions and ulcerations. Conversely, endoscopic pathognomonic features in EoE are furrows, rings, and exudates [102]. Eosinophilic infiltration is uncommon in CD esophagitis. A 14 years study conducted by the Mayo Clinic found that no esophageal biopsies in CD esophagitis showed eosinophilic infiltration [103]. Involvement of the stomach and duodenum in CD occurs in 0.5–4% of patients with CD and is usually associated with a concomitant ileal or ileo-colonic disease. The histological examination of such cases reveals non-caseating granulomas as the most common finding [104].

## 4. Overlapping Syndrome: EGIDs and IBD

**Figure 2:**
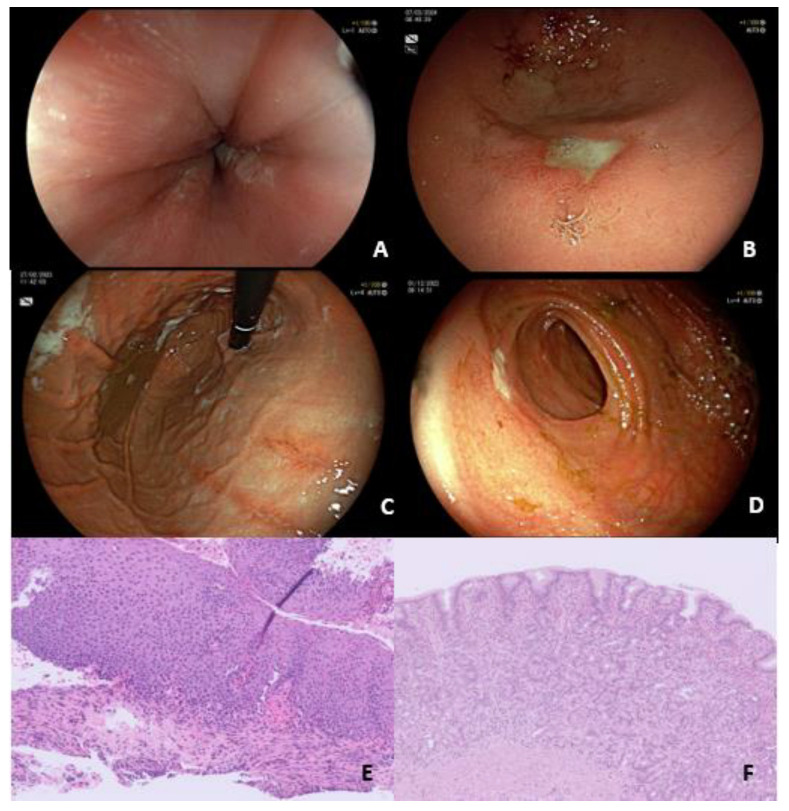
Concomitant Crohn’s disease and EGID: during colonoscopy (**D**), peri-anastomotic mild activity of IBD was documented (Rutgeerts score i2), while the esophagogastroduodenoscopy (EGD) revealed in the esophagus oedema, exudates and longitudinal furrows and erythema and linear erosions in the stomach (**A**,**C**) with multiple gastric clear-based ulcers (III, sec Forrest Classification) (**B**). Histological examination of the esophagus revealed diffuse eosinophilic infiltrate involving the squamous mucosa (200/HPF), basal zone hyperplasia, focal surface desquamation, and lamina propria fibrosis (**E**). Diffuse eosinophilic infiltrate involving the mucosa and the submucosa with intra-epithelial eosinophils, mucin depletion, reactive epithelial changes, and mild architectural distortion was observed in the gastric specimens (**F**).

Non-esophageal EGIDs are still considered rare diseases, and there is currently a lack of data from large retrospective and prospective studies about the potential for overlapping syndrome with IBD. The available data come from anecdotal case reports, which suggests a potential correlation with anti-TNF medications. For example, a 10-year-old patient with Crohn’s disease who was exposed to infliximab developed worsening abdominal pain and diarrhea, along with peripheral eosinophilia and eosinophilic infiltration in the duodenum and stomach. These symptoms improved when the patient was taken off the infliximab [105]. In a more recent case report published by Konstantinos et al. [106], a 67-year-old female patient was diagnosed with both EoC and CD in the absence of other potential precipitating factors such as infections or medications. Symptoms of EGIDs mainly involving small and/or large bowel include chronic diarrhea, while endoscopy shows diffuse erythema of the colonic mucosa with sparing of the ileal mucosa and the presence of a mixed CD/EoC pattern on histological examination. In Figure 2, we report the clinical case of a young female patient affected by ileo-colonic CD who was referred to the emergency department for anemia and emesis with blood streaks.

Regarding the overlap between IBD and EoE, Limketkai et al. [107] in a 7-year prospective study found a risk ratio of 5.4 and 3.3, respectively, for CD and UC patients developing EoE. A pre-existing diagnosis of EoE increased the risk of a new and later IBD diagnosis with similar risk ratios. Male and younger IBD patients were more likely to develop EoE, whereas among EoE patients, male gender was only associated with a higher risk of developing UC [107]. In addition, in a recent Swedish national study, Uchida et al. [9] reported a 4-fold increased risk of developing IBD in EoE patients, while IBD patients had up to 15 times increased odds of a subsequent diagnosis of EoE. Interestingly, EoE was more commonly associated with CD, although it shares type 2 inflammatory pathways with UC [9]. Similar results were found in a retrospective pediatric study, which also reported a difference in the timing of EoE diagnosis between UC and CD; while EoE was frequently diagnosed during CD follow-up, the onset of EoE was concomitant in 64.7% of UC patients [108]. Furthermore, 83% of the co-diagnoses of EoE-CD were made during CD remission or mild activity, and 10 CD patients developed EoE during clinical remission after anti-TNF therapy, supporting the hypothesis of a correlation between anti-TNF therapy and GI eosinophilia. A possible explanation may be that CD is driven by Th1 immunity, and the resulting suppression may lead to uncontrolled upregulation of the Th2 immune pathway [108,109]. In addition, the severity and extent of disease in overlapping syndromes differ from those in isolated forms. For example, histological remission of EoE with PPI treatment is estimated to be around 50.5% [110,111] and could reach up to 57.8–64.9% with STCs in two recent systematic reviews [112,113]. However, Urquhart et al. [114] found that in overlapping syndromes, eosinophilic disease had a more severe and fibro-stenotic pattern, with more than half of the patients presenting rings and strictures at the diagnosis. In more detail, the rate of histological remission after undergoing PPI and STCs was, respectively, 8.7% and 16.7% in the UC cohort and 38.7% and 31.8% in the CD cohort. Over half of the UC patients exhibited pancolitis, while 46.8% of the CD patients displayed ileo-colonic involvement. Although data are limited, no great differences in symptoms characteristics and severity and endoscopic features have been reported between patients with co-diagnosis of IBD and EoE and patients affected by EoE alone [115]. Nevertheless, some authors reported a major prevalence of dysphagia in patients with overlap syndrome and an early diagnosis of IBD [116]. The evidence basis for the clinical outcomes of co-diagnosis of IBD and EoE is limited, and the available data are not entirely consistent. In this context, Limketkai et al. [107] evaluated an elevated risk of IBD complications in co-diagnosis of IBD and EoE, with an increased likelihood of requiring systemic steroids and biological treatment. The study found a reduced risk of bowel resection in both CD and UC. Similarly, Malik et al. [117] reported in a recent retrospective cohort study that there was an increased composite risk of IBD-related complications for both CD and UC [(CD: adjusted HR (aHR) 1.14, *p* < 0.005; UC: aHR 1.17, *p* < 0.01)], as well as a need for biologic treatment for IBD. However, there were no significant differences in surgical resection and the need for systemic steroids [117]. EoE in the context of concurrent IBD was found to be significantly associated with a lower risk of food bolus impaction and a greater need for biological therapies, in comparison to non-IBD-EoE counterparts [117]. This may be attributed to the enhanced endoscopic surveillance and the concurrent administration of biological therapy in IBD treatment.

In conclusion, patients with IBD and EoE have a higher risk of developing immune-mediated comorbidities than those with only one of the two disorders. This risk is particularly high for Th1-mediated disorders, such as coeliac disease and rheumatological IBD-related conditions, and for Th2-mediated disorders such as eczema and asthma.

## 5. Treatment of IBD and Overlapping EoE

To date, there is a paucity of data regarding the treatment of IBD with concomitant EoE. The optimal treatment strategies in the setting of co-occurrence remain unknown. The majority of data on treatment outcomes, escalation, and safety derive from pediatric studies. Overall, lower clinical and histologic response rates have been observed in cases of EoE and coexisting IBD, suggesting that this phenotype might be more difficult to treat [114]. Conversely, further studies have reported that patients with IBD alone have significantly higher rates of treatment escalation and hospitalization compared to those with EoE-IBD or EoE alone [118]. Biologic therapies, such as anti-TNF agents, are commonly used to treat IBD. However, they have not proven effective in treating EoE [119], despite TNF-α being upregulated and highly expressed by esophageal epithelial cells in patients with EoE [120]. Their role is controversial; as previously stated, TNF-alpha inhibitors have been associated with a higher risk of developing eosinophilic disorders in IBD patients [105,106]. However, isolated cases of steroid-dependent EoE have been reported and successfully treated with other anti-TNFα such as adalimumab, mainly in the pediatric population [121,122]. Indeed, a retrospective case–control study including pediatric patients demonstrated that the use of anti-TNFα in managing pre-existing IBD provided protection against EoE development (RR 0.314, 95% CI 0.159–0.619) [123].

Moreover, another biological drug that may be employed in the treatment of overlapping conditions is represented by vedolizumab, an anti-α4β7 integrin agent that inhibits leukocyte trafficking in CD and CU. This drug has recently been reported for the treatment of refractory EoE [124]. In detail, clinical response, along with endoscopic and histological improvement, was observed in patients with lower gastrointestinal tract eosinophilic involvement and with eosinophilic duodenitis [125,126]. In support of the hypothesis that the two diseases operate through mechanisms of shared leukocyte trafficking, a number of studies have shown that the presence of mucosal eosinophilia is an independent predictor of a higher rate of efficacy of vedolizumab in IBD [127,128].

As previously mentioned, EoE is typically characterised by a Th2 inflammatory response, whereas IBD, especially CD, involves a Th1/Th17 response with the participation of interleukin 10 (IL-10). Nevertheless, both disease states exhibit shared cytokine and T-helper cell-mediated mechanisms. In CD, there is an elevation in mucosal IL-5 expression, while UC exhibits increased eotaxin expression, which acts as a chemoattractant for eosinophils. IL-5 has been linked to eosinophil activation in tissues and may contribute to early mucosal damage in CD [107,129]. This suggests that EoE and IBD may share similar biological pathways and therapeutic targets but may lack a definitive common inflammatory pathway that prevents a full response in patients with the two overlapping conditions. Dupilumab, a monoclonal antibody that blocks interleukin-4 (IL-4) and IL-13 and the only biologic approved for the treatment of EoE, has not been studied in patients with IBD and concomitant EoE. Nevertheless, dupilumab has been demonstrated to be safe and efficacious in the treatment of atopic dermatitis among patients with IBD, including primary atopic dermatitis and dermatitis triggered or exacerbated by anti-TNF therapy [130,131]. A Phase 2 clinical trial is currently enrolling participants to assess the efficacy and safety of dupilumab therapy in patients with UC with an eosinophilic phenotype [132]. Finally, combination treatment with monoclonal antibodies targeting hyper-eosinophilic syndrome in overlapping EoE and IBD syndrome has been reported without safety concerns [133].

## 6. Discussion

**Figure 3:**
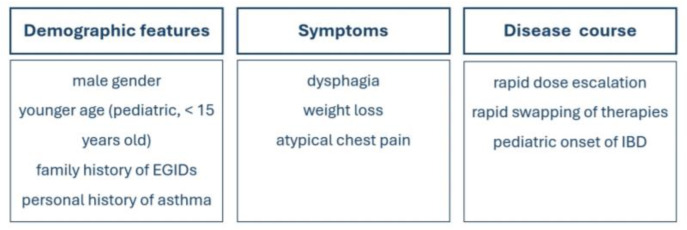
Red flags for suspecting and ruling out EGIDs in cases of known IBD.

IBD, EoE, and non-EoE EGIDs are examples of gastrointestinal diseases characterized by underlying immune dysregulation. Current research is making a significant effort to understand their shared pathogenic pathways. Preclinical and clinical data indicate and support that these disorders hold common genetic and early environmental factors. The association of IBD and EGIDs appears to be bidirectional. Indeed, recent nationwide cohort studies have reported a nearly 4-fold increased risk of later IBD diagnosis in patients with established EoE and 15-fold increased odds of later EoE diagnosis in patients with established IBD [9]. The diagnosis of an overlapping IBD and EGID poses several challenges for physicians; recognizing possible ‘red flags’ (disease-specific signs and symptoms) of an adjunctive inflammatory disorder in the context of an established disease with a known GI involvement may be tricky and delay further diagnostic work-up and referral. Secondly, medications used for the underlying chronic disease may confound the onset of the later IBD/EGIDs, and finally, some patients might have an initial misclassification of the first IBD/EGID. Regarding IBD, after many years of collaborative research across multiple societies, so-called ‘red flags’ have been identified and standardized for the appropriate referral of patients to specialists. This process has not yet been initiated for associations with concurrent, even rarer, conditions such as EGIDs. In Figure 3, we propose an algorithm for suspecting and ruling out EGIDs in case of known IBD. The occurrence of extra-intestinal symptoms (i.e., dysphagia, weight loss, rapid dose escalation or swapping of therapies) requires further investigation in IBD patients. The preliminary characterization of coexistent IBD and EGIDs, particularly EoE, has shown that these patients may present accelerated organ remodeling (i.e., esophageal rings and strictures), displaying a modified phenotype and natural history [114]. Coexistent IBD and EGIDs may configurate a sub-set of yet unidentified difficult-to-treat patients. Preclinical data have shown clinical efficacy and achievement of therapeutic goals in both conditions selectively blocking the IL-4, IL-5 and IL-13 pathways. Still, a precise characterization of these molecular pathways, especially that of Th-2 inflammation (which is involved in the creation of organ damage) is necessary to identify and develop new therapeutic targets. In our view, to fully comprehend the complex relationship between IBD and EGIDs, larger studies incorporating genetic and environmental data are needed. Awareness of and education on coexisting IBD and EGIDs must be promoted by healthcare professionals and require multidisciplinary management.

## Data Availability

Data sharing is not applicable. No new data were created or analyzed in this study.
